# Comprehensive assessment of neurocognitive function, inflammation markers, and adiposity in treated HIV and control

**DOI:** 10.1097/MD.0000000000031125

**Published:** 2022-10-21

**Authors:** Christian Mouchati, Vanessa El Kamari, Abdus Sattar, Jiao Yu, Grace A McComsey

**Affiliations:** a Case Western Reserve University, School of Medicine, OH, USA; b University Hospitals Cleveland Medical Center, Cleveland, OH, USA; c Department of Pediatrics, Case Western Reserve University, Cleveland, OH, USA; d Rainbow Babies and Children’s Hospital, Cleveland, OH, USA.

**Keywords:** antiretroviral therapy, HIV pathogenesis, inflammation, neurological/brain

## Abstract

To compare the neurocognitive scores between persons living with human immunodeficiency virus (PLWH) and persons without human immunodeficiency virus (HIV) and assess the relationship between neurocognition, HIV status and variables, inflammation, and body composition measures. Cross-sectional study involving 225 participants (126 PLWH on antiretroviral therapy [ART] and 99 persons without HIV). For the first time in HIV, we used Cognivue®, an food and drug administration (FDA)-approved computer-based test to assess cognitive function. The test was calibrated to individuals’ unique cognitive ability and measured 6 cognitive domains and 2 performance parameters. Markers of inflammation, immune activation, insulin resistance, and body fat composition (using dual-energy X-ray absorptiometry scan) were collected. Classical *t* tests, chi-square tests, and spearman correlations were used to compare and explore relationships between variables. Inverse probability weighting adjusted average treatment effect models were performed to evaluate the differences between PLWH and persons without HIV, adjusting for age, race, sex, and heroin use. Overall, 64% were male, 46% were Black, with a mean age of 43 years. Among PLWH, 83% had an undetectable HIV-1 RNA level (≤20 copies/mL). Compared persons without HIV, PLWH performed poorer across 4 domains: visuospatial (*P* = .035), executive function (*P* = .029), naming/language (*P* = .027), and abstraction (*P* = .018). In addition, PLWH had a significantly longer processing speed time compared to controls (1686.0 ms vs 1606.0 ms [*P* = .007]). In PLWH, lower cognitive testing domain scores were associated with higher inflammatory markers (high sensitivity C-reactive protein [hsCRP]) and with higher total fat and visceral adipose tissue (*P* < .05). Neurocognitive impairment (NCI) in HIV is associated with inflammation and total and central adiposity.

## 1. Introduction

With the use of antiretroviral therapy (ART), persons living with human immunodeficiency virus (PLWH) are presumed to reach a life expectancy approaching population norms.^[1]^ Despite therapeutical advances, patients still suffer from human immunodeficiency virus (HIV)-associated neurocognitive disorders (HAND) described since the start of the HIV epidemic.^[2]^ According to a recent meta-analysis, the overall prevalence of HAND was 43%.^[3]^ With therapeutical advances, the incidence of HIV-associated Dementia has significantly decreased (to a prevalence of 5%),^[4]^ and the incidence of Asymptomatic Neurocognitive Impairment (NCI) has dramatically increased (with a prevalence of 23%), with the increase of life expectance of the HIV population.^[5]^ Patients with asymptomatic NCI are unaware of their performance and perceptual processing decline, usually revealed by neuropsychological testing.^[6]^

Many tests are being used for the assessment of HIV dementia like the Montreal cognitive assessment, the mini-mental state examination, the Simioni symptom questions, and the cognitive assessment tool-rapid version.^[7–11]^ However, to our knowledge, there isn’t any conventional testing method validated for the screening and classification of the different HAND subtypes.^[12]^ Indeed, despite its widespread adoption, mini-mental state examination was deemed inefficient for diagnosing HAND.^[13]^

Compared to paper and pencil tests, *Cognivue©* could be a more accessible tool by surmounting the limitations of traditional testing like educational, language, gender, and cultural bias,^[14,15]^ and subjectivity.^[16]^ The *Cognivue©* is a 10-minute computerized cognitive self-test that measures the cognitive function across 6 cognitive domains: visuospatial, executive function/attention, naming/language, memory, delayed recall and abstraction and 2 speed performance parameters: reaction time and speed processing.

HAND mechanism is still poorly understood, thus the need to further study the link between HIV-associated neurocognitive decline and inflammation, which is suspected to be one of the pillars of cardiovascular disease and metabolic dysfunction. Exploring the different associations between inflammation and the stated conditions could hold the key to unlocking a better understanding of their pathophysiology, leading to improved management of HIV complications and other cardiometabolic diseases. Therefore, this study aimed to observe the association between PLWH neurocognitive performance and the different demographic characteristics and inflammatory markers, body composition measures, and metabolic markers compared to persons without HIV.

## 2. Methods

### 2.1. Study design/population

This is a cross-sectional study measuring neurocognitive scores in PLWH and persons without HIV adults with concomitant assessment of HIV variables and measurements of body composition and inflammatory markers. Data, for both study arms, was obtained from participants older than 18 years who underwent testing in the same settings during their entry visit in Institutional review board approved metabolic studies conducted at the Metabolic Research Center at University Hospitals Cleveland Medical Center from December 2019 to May 2021. For the PLWH group, participants were over 18 years old with a documented HIV-1 infection and on the same ART regimen for more than 6 months prior to enrollment. Before testing, participants were asked by the examiners about the following exclusion criteria: current neurologic symptom or any past or current documented neurologic disease (e.g., stroke, neurodegenerative disorder, encephalopathy, and dementia). Participants were also asked to report any medication that could affect the study results or any documented acute illness, malignancy, or inflammatory condition within 30 days before recruitment; if meeting any of these criteria, participants were excluded. Before any testing was performed, participants signed a written informed consent in their respective Institutional review board-approved studies.

### 2.2. Study assessments

#### 2.2.1. Medical history and demographics.

Demographics, social history, and substance use were collected through face-to-face interviews performed by a trained health care professional. Medical records were used for the PLWH group after obtaining permission to verify elements of the medical history, such as detailed past and current ART and non-ARV medication use, CD4 + T-cell count, HIV viral load (VL), and diagnosis date if known to calculate HIV duration.

#### 2.2.2. Neurocognitive assessment.

Neurocognitive performance was evaluated using an FDA-cleared computerized testing tool called Clarity by *Cognivue*® (FDA De Novo Number 130033) validated against the Saint Louis University Mental Status exam to detect early indicators of cognitive impairment.^[17]^ Participants were asked to answer a series of questions on a computer monitor for 10 minutes.^[18]^ (See Image, Supplemental Content 1, http://links.lww.com/MD/H608, which illustrates the Cognivue Clarity device). A score ranging from 0 to 100: the *cognivue* clinical score, also called average neurocognitive score, was calculated where a high risk of cognitive impairment corresponds to ≤50, and no risk of impairment to ≥75.^[18,19]^ A 2-page report was generated presenting an overall score, with a subsequent breakdown into the 6 following cognitive domains: visuospatial, executive function, naming/language, memory, delayed recall, and abstraction. On the second page, 2 additional important parameters were recorded: Reaction Time and Speed Processing Time. (See Images, Supplemental Content 2, http://links.lww.com/MD/H609 and 3, http://links.lww.com/MD/H610, which illustrates an example of the Cognivue Clarity report).

#### 2.2.3. Inflammation, monocyte activation, and gut integrity markers.

Blood was stored at −80°C and batched until processing without prior thaw. Enzyme-linked immunosorbent assay assays were performed to measure markers of systemic inflammation (soluble tumor necrosis factor receptors I and II, high sensitivity C-reactive protein [hsCRP], interleukin 6 [IL-6] [R &D Systems, Minneapolis, Minnesota]), coagulation:d-dimer (Diagnostica Stago, Parsippany, New Jersey), and oxidized low-density lipoprotein assays (Uppsala, Mercodia, Sweden), as well as markers of monocyte activation soluble CD14 and CD163 (R&D Systems, Minneapolis, Minnesota).^[20–23]^ The soluble intercellular adhesion molecule 1 was measured by Enzyme-linked immunosorbent assays (R&D Systems, Minneapolis, Minnesota).^[24]^ A marker of gut permeability: zonulin-1 (Promocell Germany), a marker of microbial translocation (Levels of lipopolysaccharide-binding protein [LBP, Hycult Biotech Inc. Pennsylvenia]), and a marker of fungal translocation β-D-glucan (Mybiosource Inc. California) were measured to assess gut-barrier integrity.^[25–27]^

#### 2.2.4. Body composition measures.

Glucose and insulin were measured on blood samples drawn after at least 8 hours of fasting. The homeostatic model assessment of insulin resistance (HOMA-IR) was calculated based on the following equation: log_10_ (HOMA-IR) = log_10_ (glucose (mg/dL) × log_10_ (insulin (IU)/405). We considered HOMA-IR > 2.5 as the threshold for insulin resistance.^[28]^

Total, limb, and trunk fat, as well as lean body mass, were quantified radiologically using whole body dual-energy X-ray absorptiometry scan. Abdominal total adipose tissue, visceral adipose tissue (VAT), and subcutaneous adipose tissue were measured by non contrast computed tomography scan, using a single slice imaging at the level of the L4-L5 to optimize measurement accuracy and minimize radiation.^[29]^

### 2.3. Statistical analysis

We checked data quality and distributions using frequency analysis, descriptive statistics, and graphs. Covariates of interest are log-transformed for meaningful interpretation of regression coefficients (see below on regression analysis). The differences in study variables between the PLWH and the persons without HIV were evaluated using Fisher’s exact tests and Wilcoxon tests, as appropriate. We used fitted inverse probability weighting adjustment model to compare neuro-cognitive scores between the PLWH and the persons without HIV while adjusting for baseline characteristics and drug use.

Spearman correlations were used to compare and explore relationships between neurocognitive scores and body composition measures. Due to the skewness of the neurocognitive scores, 2 quantile regression analyses models were used to evaluate the associations between neurocognitive scores and inflammatory markers in PLWH and persons without HIV (each in a separate model), adjusting for age, sex, race, and heroin use. Inflammatory markers were log-transformed.

Statistical significance is defined with a *P* value <.05. All tests are 2-sided. The statistical analyses were performed using software Stata 15.0 and R 3.4.1.

## 3. Results

### 3.1. Subject characteristics

A total of 225 participants were enrolled, including 126 PLWH and 99 persons without HIV. Overall, 64% were male, and 54% were Non-Hispanic Whites, with a median age of 43 years. At baseline, PLWH were older and predominantly black males compared to persons without HIV. Overall, 54% of all participants were current smokers, 62% reported current alcohol consumption, 39% reported current use of marijuana, 13% using cocaine, and 20% using heroin. Participants’ baseline characteristics are presented in Table 1.

**Table 1:** Baseline characteristics of study participants by assigned treatment group.

	Overall median [IQR]/Frequency (%)*	HIV- median [IQR]/Frequency (%)*	HIV + median [IQR]/Frequency (%)*	*P*†
N	225	99	126	
Age (yrs)	42.89 [33.72, 56.20]	38.19 [32.90, 47.32]	50.75 [34.59, 59.02]	**<.001**
Male (%)	145 (64.4)	52 (52.5)	93 (73.8)	**.002**
White (%)	122 (54.2)	81 (81.8)	41 (32.5)	**<.001**
Non-Hispanic/Latino (%)	204 (90.7)	90 (90.9)	114 (90.5)	1
BMI (kg/m^2^)	28.13 [23.65, 32.83]	27.43 [22.89, 31.73]	28.44 [24.66, 33.32]	.147
Smoking (%)	121 (53.8)	63 (63.6)	58 (46.0)	**.031**
Alcohol (%)	139 (61.8)	57 (57.6)	82 (65.1)	.283
Marijuana use (%)	88 (39.1)	33 (33.3)	55 (43.7)	**.015**
Cocaine use (%)	30 (13.3)	17 (17.2)	13 (10.3)	**.045**
Heroin use (%)	44 (19.6)	33 (33.3)	11 (8.7)	**<.001**
Undetectable VL (%)	100 (82.6)		100 (82.6)	
CD4 (cell/mm^3^)	738.00 [525.50, 1021.00]		738.00 [525.50, 1021.00]	
IL-6 (pg/mL)	2.87 [1.77, 5.07]	3.07 [1.89, 5.51]	2.70 [1.71, 4.54]	.377
sTNFR-I (pg/mL)	1163.80 [980.67, 1524.00]	1267.31 [1009.06, 1551.69]	1135.44 [970.97, 1463.71]	.393
sTNFR-II (pg/mL)	2610.67 [1948.10, 3428.37]	2772.03 [2087.39, 3760.54]	2428.26 [1867.07, 3147.88]	**.029**
hsCRP (ng/mL)	3681.32 [1289.54, 8999.07]	4055.49 [1602.50, 9962.15]	2834.87 [1034.38, 8072.97]	.112
sICAM (ng/mL)	281.64 [159.84, 379.29]	464.78 [386.17, 555.28]	251.38 [153.29, 373.72]	**.005**
LBP (ng/mL)	20272.02 [15147.86, 27874.30]	19248.84 [15152.81, 26611.03]	20351.55 [15083.35, 29256.95]	.418
D-dimer (ng/mL)	430.88 [248.69, 703.55]	409.35 [243.58, 706.22]	448.70 [257.14, 697.06]	.787
Zonulin (ng/mL)	3.84 [3.03, 4.53]	4.09 [3.29, 4.78]	3.74 [2.87, 4.27]	**.017**
BDG (pg/mL)	375.46 [225.51, 473.87]	421.31 [344.01, 517.26]	262.14 [193.27, 396.34]	**<.001**
HOMA-IR	2.37 [1.39, 3.57]	2.27 [1.50, 3.26]	2.42 [1.32, 3.81]	.572
Total LBM (g)	54268.65 [44267.58, 62309.02]	49567.15 [43398.35, 58598.80]	56950.40 [47087.55, 63642.85]	**.011**
Trunk fat (kg)	13774.30 [8819.93, 19304.45]	11891.75 [8351.02, 16739.50]	15042.90 [10659.85, 20319.67]	**.012**
SAT area (cm^2^)	297.70 [187.65, 362.50]	281.85 [183.10, 345.53]	301.80 [210.50, 366.80]	.48
VAT area (cm^2^)	115.20 [79.20, 159.60]	91.70 [65.35, 126.95]	123.20 [94.00, 176.35]	**.005**

Bold font indicates statistical significance (*P* < 0.05).

*All continuous variables are summarized as median [1st quantile, 3rd quantile].

†The Wilcoxon rank-sum test is used for continuous variables. Fisher’s exact tests are used for categorical variables.

% = percent, BDG = β-D-glucan, BMI = body mass index, CD4 = cluster of differentiation 4, cell/mm^3^ = cell per millimeter cube, cm^2^ = centimeter square, D-dimer = Domain dimer, g = gram, HOMA-IR = Homeostatic Model Assessment of Insulin Resistance, hsCRP = high sensitivity C-Reactive Protein, IL-6 = Interleukin 6, kg/m^2^ = kilogram per meter square, IQR = interquartile range, LBM = Lean Body Mass, LBP = lipopolysaccharide-binding protein, N = number of participants, ng/mL = nanogram per milliliter, pg/mL = picogram per liter, SAT = subcutaneous adipose tissue, sICAM = soluble Intercellular Adhesion Molecules, sTNFR-I = soluble Tumor Necrosis Factor receptors I, sTNFR-II = soluble Tumor Necrosis Factor receptors II, VAT = visceral adipose tissue, VL = viral load.

### 3.2. Neurocognitive testing

Overall, the median *cognivue* clinical score was 77.00 (interquartile range, 64.00–86.00). PLWH had a lower median *cognivue* clinical score compared to persons without HIV (75.5 vs 80; *P* = .058). When looking at the 6 individual domains, PLWH had a significant lower performance across the following 4 domains: visuospatial (78.0 vs 85.0 [*P* = .035]), executive function (74.0 vs 79.0 [*P* = .029]), naming/language (79.5 vs 82.0 [*P* = .027]), and abstraction (80.0 vs 84.0 [*P* = .018]). When studying performance testing of persons without HIV, a significantly longer median processing time was recorded compared to healthy individuals (1686.0 ms vs 1606.0 ms [*P* = .007]) (Table 2).

**Table 2:** Descriptive statistics of neurocognitive scores.

	Overall median [IQR]/Frequency (%)*	HIV- median [IQR]/Frequency (%)*	HIV + median [IQR]/Frequency (%)*	*P*†
n	225	99	126	
Visuospatial	81.00 [68.00, 89.00]	85.00 [71.50, 90.00]	78.00 [65.25, 88.00]	.035
Executive	76.00 [64.00, 85.00]	79.00 [66.50, 87.00]	74.00 [60.00, 84.00]	.029
Memory	77.00 [61.00, 90.00]	78.00 [63.50, 90.00]	76.00 [59.25, 89.00]	.363
Naming/language	81.00 [66.00, 89.00]	82.00 [71.50, 90.00]	79.50 [62.25, 88.00]	.027
Delayed recall	83.00 [67.00, 92.00]	86.00 [74.00, 92.50]	81.00 [64.25, 92.00]	.12
Abstraction	82.00 [65.00, 92.00]	84.00 [70.00, 94.00]	80.00 [62.25, 89.00]	.018
Visual salience reaction time (ms)	801.00 [652.00, 1055.00]	833.00 [683.50, 1045.50]	784.50 [645.00, 1053.50]	.233
Adaptive motor control reaction time (ms)	519.00 [455.00, 607.00]	505.00 [447.00, 582.50]	528.00 [461.00, 636.75]	.102
Average speed processing time (ms)	1650.00 [1523.00, 1789.00]	1606.00 [1498.00, 1720.00]	1686.00 [1547.00, 1818.00]	.007
Average neurocognitive score	77.00 [64.00, 86.00]	80.00 [66.00, 89.00]	75.50 [61.00, 86.00]	.058

*All continuous variables are summarized as median [1st quantile, 3rd quantile].

†The Wilcoxon rank-sum test is used for continuous variables. Fisher’s exact tests are used for categorical variables.

IQR = interquartile range, ms = milliseconds.

When comparing to the persons without HIV group, after adjustment for age, sex, and race, the PLWH group was estimated to have respectively 6.34 times (*P* = .03) and 6.69 times (*P* = .04) higher likelihood of low executive function scores and naming/language score (Table 3).

**Table 3:** Average treatment effects of study groups on neuro-cognitive scores using inverse probability weighting adjustment.

Outcomes	ATE*	Robust S.E.	*P*
**Visuospatial**	2.29	3.23	.48
**Executive function**	-6.34	2.96	**.03**
**Memory**	-3.41	2.52	.18
**Naming/language**	-6.69	2.89	**.02**
**Delayed recall**	-3.28	2.28	.15
**Abstraction**	-4.69	2.74	.08
**Visual salience reaction time**	-375.14	241.34	.12
**Adaptive motor control reaction time**	-53.55	36.04	.13
**Average speed processing time**	-5.07	49.68	.92
**Average neurocognitive score**	-3.21	2.22	.15

Inverse-probability weighting (IPW) adjusted average treatment effect models are performed to evaluate the differences between HIV-infected and control groups, adjusting for age, race, sex, marijuana use, cocaine use, and heroin use.

* ATE = average treatment effect.

### 3.3. Markers of inflammation and body composition

Markers of systemic inflammation were not increased in PLWH; in fact, 2 markers were lower in the PLWH group; soluble tumor necrosis factor receptors II levels (*P* = .029) and ICAM (*P* = .005). Similarly, Zonulin was lower in the PLWH group (*P* = .017). Concerning the body composition’s indices, the PLWH had a higher VAT area than persons without HIV (*P* = .005). PLWH had higher lean body mass index and trunk fat compared to persons without HIV (*P* = .011; *P* = .012, respectively). However, there was no statistically significant difference between insulin resistance calculated using the homeostatic model HOMA-IR in PLWH and persons without HIV (2.42 [1.32, 3.81], 2.27 [1.50, 3.26], respectively).

### 3.4. Relationships between neurocognitive scores and HIV related variables of interest

**Figure 1.**
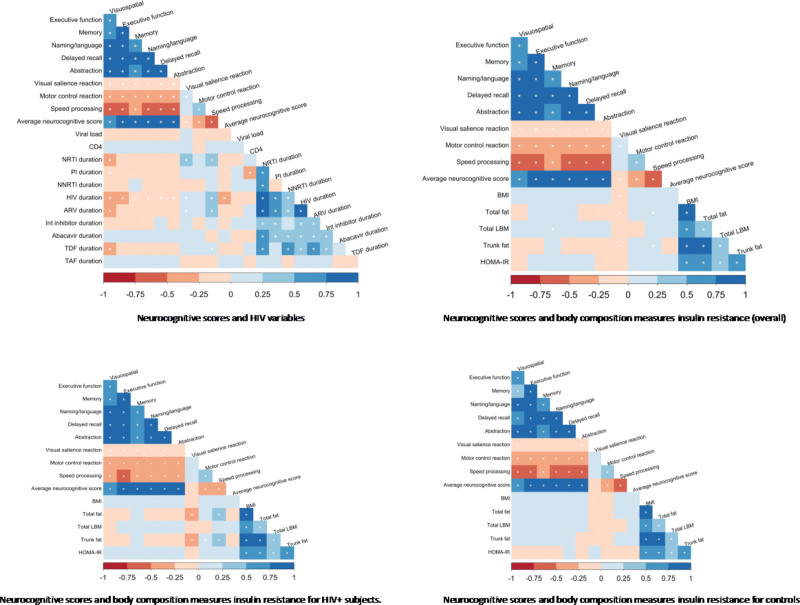
Spearman correlations between the Neurocognitive scores and the variables of interest.

Correlations between neurocognitive scores and HIV-related variables are presented in Figure 1. Longer duration of HIV infection was significantly correlated with lower neurocognitive scores (average score, executive function, memory, naming/language, delayed recall, and abstraction) and longer visual salience reaction and speed processing time (as seen in Fig. 1). No significant associations were observed between CD4 + T cells count and HIV VL.

Antiretroviral duration and nucleoside reverse transcriptase inhibitors (NRTIs) duration were negatively correlated with visuospatial scores, but they were positively correlated with longer visual salience reaction and speed processing time. Also, Tenofovir Disoproxil Fumarate duration demonstrated a negative correlation with visuospatial performance and a positive correlation with speed processing time (*P* < .05). Protease Inhibitors were also negatively correlated with visual salience (*P* < .05). No other correlation was found between the different neurocognitive scores and the rest of the ARV medications.

### 3.5. Relationship between neurocognitive scores and inflammatory and gut markers

The quantile regression analyses models adjusting for age, sex, race, and heroin use, indicate that the association between neurocognitive scores and inflammatory markers was prominent in the PLWH group. Lower cognitive testing domain scores (visuospatial, memory, delayed recall, [all *P* < .05]) were associated with higher hsCRP, but not other inflammation markers, among the PLWH group only (Table 4). None of the measured gut markers were associated with neurocognitive scores.

**Table 4:** Quantile regression of neurocognitive scores and inflammatory markers among HIV-infected and controls.

Outcomes	IL6		sTNFR-I		sTNFR-II		hsCRP	
	**Coef.(β**)†	***P***	**Coef.(β**)†	***P***	**Coef.(β**)†	***P***	**Coef.(β**)†	***P***
Controls								
Visuospatial	-0.023	.992	-0.877	.882	-0.677	.872	0.955	.474
Executive function	0.926	.707	5.085	.414	4.823	.310	1.161	.412
Memory	2.711	.361	1.505	.847	3.137	.590	-0.528	.777
Naming/language	-0.136	.953	5.186	.432	3.358	.499	0.830	.588
Delayed recall	1.349	.586	3.474	.597	4.103	.435	0.487	.739
Abstraction	1.738	.553	2.882	.715	3.617	.557	1.325	.474
Visual salience reaction time	17.296	.795	-113.382	.521	3.946	.974	18.092	.580
Adaptive motor control reaction time	15.531	.441	-14.390	.800	-11.058	.807	6.021	.628
Average speed processing time	-15.613	.614	-32.372	.691	-33.839	.571	-5.855	.758
Average neurocognitive score	0.908	.716	5.916	.360	3.66	.470	0.686	.631
HIV-infected								
Visuospatial	-0.795	.706	-1.123	.812	-0.650	.868	**-2.308** *	.047
Executive function	-0.071	.978	2.343	.715	1.440	.777	-1.474	.376
Memory	-0.492	.878	7.816	.270	1.137	.849	**-5.679** **	.003
Naming/language	-0.125	.969	-0.433	.961	-0.936	.885	-0.725	.702
Delayed recall	-6.248	.075	-11.209	.159	-6.422	.309	**-6.206** **	.002
Abstraction	-1.476	.671	-8.733	.225	-7.271	.249	-4.140*	.023
Visual salience reaction time	16.408	.704	-172.272	.074	-78.254	.273	14.421	.543
Adaptive motor control reaction time	-11.823	.631	-41.473	.371	-27.303	.507	-9.658	.456
Average speed processing time	40.750	.242	-32.228	.655	44.244	.473	16.362	.462
Average neurocognitive score	0.452	.884	0.994	.887	-0.569	.923	-2.232	.228

† Coefficients from quantile regression analysis for the outcome of interest, adjusting for age, sex, race, heroin use, and viral load. Inflammatory markers are log-transformed.

sTNFR-I = soluble tumor necrosis factor receptors I, sTNFR-II = soluble tumor necrosis factor receptors II

* *P < .1*.

** *P < .05.*

### 3.6. Relationship between neurocognitive scores and body composition and metabolic measures

When considering all participants, regardless of HIV status, higher body fat content (total percent fat and trunk fat), and Body Mass Index were associated with lower visual salience reaction time (*P* < .05) (Fig. 1). The same results were found when considering the PLWH group only, whereas no correlation was found within the persons without HIV group (Fig. 1).

## 4. Discussion

In this study, we used a novel tool to assess neurocognitive performance in HIV. We evaluated the relationship of the generated scores with HIV disease-specific factors, ART-specific factors, inflammatory markers, and metabolic markers, including objective measurements of regional body fat. The PLWH group had lower scores in several domains, including visuospatial, executive function, naming/language, and abstraction, and a longer speed processing time. Interestingly, our group had previously reported similar results using traditional neurocognitive assessments in a group of children and young adults with HIV; in that group, we reported lower speed processing, attention, executive functioning, expressive language, memory, visuospatial, and motor skills when compared to a control group.^[30]^

As expected, longer HIV duration was correlated with lower *Cognivue* clinical score, visuospatial, executive function, memory, naming/language, delayed recall, and abstraction scores. HIV duration and ARV duration (especially NRTI) were associated with a longer visual salience reaction, and speed processing time. ARV duration and NRTI (especially Tenofovir Disoproxil Fumarate) were also associated with worse visuospatial scores. Similarly, other studies concluded that longer HIV duration and ARV exposure (which could be only a marker of longer infection) contributes to NCI.^[31,32]^

In this study, the Food and Drug Administration (FDA)-cleared computer-based adaptive test C*ognivue* demonstrated cognitive dysfunction in PLWH. *Cognivue* is a precise, objective, time- and resource-saving reproducible test. By generating adaptive content, this tool adjusts for educational and socioeconomic bias and avoids traditional memorization issues caused by the practice effect seen in other testing modalities.^[18,33]^ Our results mirror those of a study conducted on patients with multiple sclerosis, whose cognitive scores were significantly lower compared to controls. This project paves the way for further use of C*ognivue* as an assessment tool for neurocognitive changes, especially in HIV, including in interventional clinical trials.

Regarding body composition measurements, when compared to persons without HIV, the PLWH group had significantly higher Body Mass Index, lean body mass index, VAT trunk fat, signaling overall and central obesity, an expected finding based on the current knowledge.^[34]^ Higher body fat content (total and trunk fat) in PLWH was associated with lower visual salience reaction time. Measurement of central obesity is considered reflective of a more consequential adiposity in PLWH due to a more deleterious effect of visceral adiposity in metabolic consequences like obesity.^[34]^ Our study is consistent with a study by McCutchan et al that found that higher waist circumference was associated with a higher risk of NCI in PLWH, which may be due to white matter hyperintensities and hippocampal atrophy.^[33,35]^ The mechanism of adiposity effect on neurocognitive performance is still unclear but was associated with axonal and myeline injuries and inflammation.^[36]^ Moreover, abdominal adiposity is associated with higher levels of hsCRP, a marker of systemic inflammation.^[37]^ Indeed, in our study, we found that among the PLWH, higher hsCRP was associated with lower visuospatial, memory, and delayed recall scores, signaling the potential pathway by which central obesity may lead to NCI.

Adult neurogenesis is decreased by chronic neuroinflammation from HIV-1.^[38,39]^ Although sTNF-RII is correlated with worse prognosis, ICAM with worse vascular function, and a higher level of Zonulin with mortality in PLWH, we found higher levels of sTNF-RII, ICAM, and Zonulin in the persons without HIV group compared to the PLWH group.^[24,40,41]^ This could be due to the sample selected, but future larger studies need to confirm these findings.

There are a few limitations to our study. Despite the strength of our analysis, causality cannot be demonstrated using a cross-sectional study. Although we excluded participants with acute inflammatory state and only included participants on ART; 97.52% of PLWH had HV-1 RNA < 400 copies/mL, and the highest VL was 1840 copies/mL. PLWH were much less likely to be White, which can influence results since population normative data may be less well developed (and therefore less accurate) in racial and ethnic minority groups than in majority populations. Additionally, people without HIV were more likely to use tobacco, heroin, and cocaine, which could be a conservative bias. Accounting for these multiple differences in multivariable models will absorb variance in the outcomes, which could partly explain why so few biomarkers were associated with cognitive performance. Also, more publications using *Cognivue* in the upcoming years would strengthen its reliability since it has only been compared to a single neuropsychological test and has not yet been validated in PLWH.^[18]^ A longitudinal follow-up using *cognivue* would have also helped better explore the effect of HIV and ARV duration on neurocognitive performance. Further studies are needed to better assess the effect of HIV on the brain testing scores and how these scores affect each other.

In conclusion, our results may have a significant clinical relevance, suggesting that using a novel, easy to administer software technology, PLWH had lower visuospatial, executive function, naming/language, abstraction, and processing speed functions compared to persons without HIV. Also, HIV duration, ARV duration, and total and central body fat measures were negatively associated with visual salience and speed processing. Higher HsCRP, but not gut markers or other inflammation markers, was correlated with worse visuospatial, memory, and delayed recall function. Further studies could consolidate the aspects of the involvement of chronic inflammation on neurocognitive function and assess the value of Cognivue in longitudinal studies of PLWH.

## Author contributions

C.M., V.E.K., A.S., J.Y., and G. A. M. contributed to study concept and design. All authors contributed to the acquisition of data A.S., J.Y., and G. A. M. contributed to analysis and interpretation of data. C. M., V.E.K., and G. A. M. drafted the manuscript. All authors contributed to the critical revision of the manuscript for important intellectual content. A.S. and J. Y. contributed to statistical analysis. G. A. M. obtained funding. C. M. and V.E.K. contributed to administrative, technical, or material support. G. A. M. supervised the study.

**Conceptualization:** Vanessa El Kamari, Grace A Mccomsey.

**Data curation:** Grace A Mccomsey.

**Formal analysis:** Abdus Sattar, Jiao Yu, Grace A Mccomsey.

**Funding acquisition:** Grace A Mccomsey.

**Investigation:** Vanessa El Kamari, Grace A Mccomsey.

**Methodology:** Grace A Mccomsey.

**Resources:** Grace A Mccomsey.

**Supervision:** Grace A Mccomsey.

**Validation:** Christian Mouchati.

**Writing – original draft:** Christian Mouchati, Grace A Mccomsey.

**Writing – review & editing:** Christian Mouchati, Vanessa El Kamari, Abdus Sattar, Jiao Yu, Grace A Mccomsey.

## Supplementary Material


