# Supplementary figures and images for: Comprehensive assessment of neurocognitive function, inflammation markers, and adiposity in treated HIV and control
Source: Medicine (Baltimore). 2022 Oct 21;101(42):e31125. doi: 10.1097/MD.0000000000031125 (PMC9592384; doi:10.1097/MD.0000000000031125)

Supplemental digital content 1: Image that illustrates the Clarity Cognivue machine.

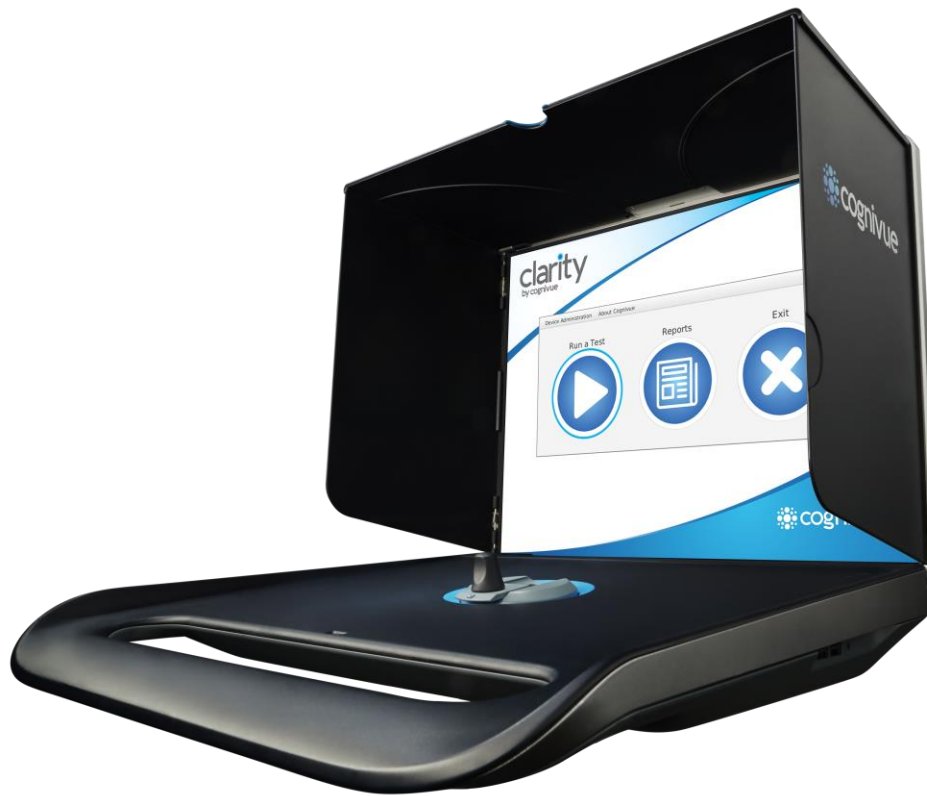

Supplement: Supplementary file 1 [file medi-101-e31125-s001.pdf]

Supplemental digital content 2: Image that illustrates the Clarity Report page 1.

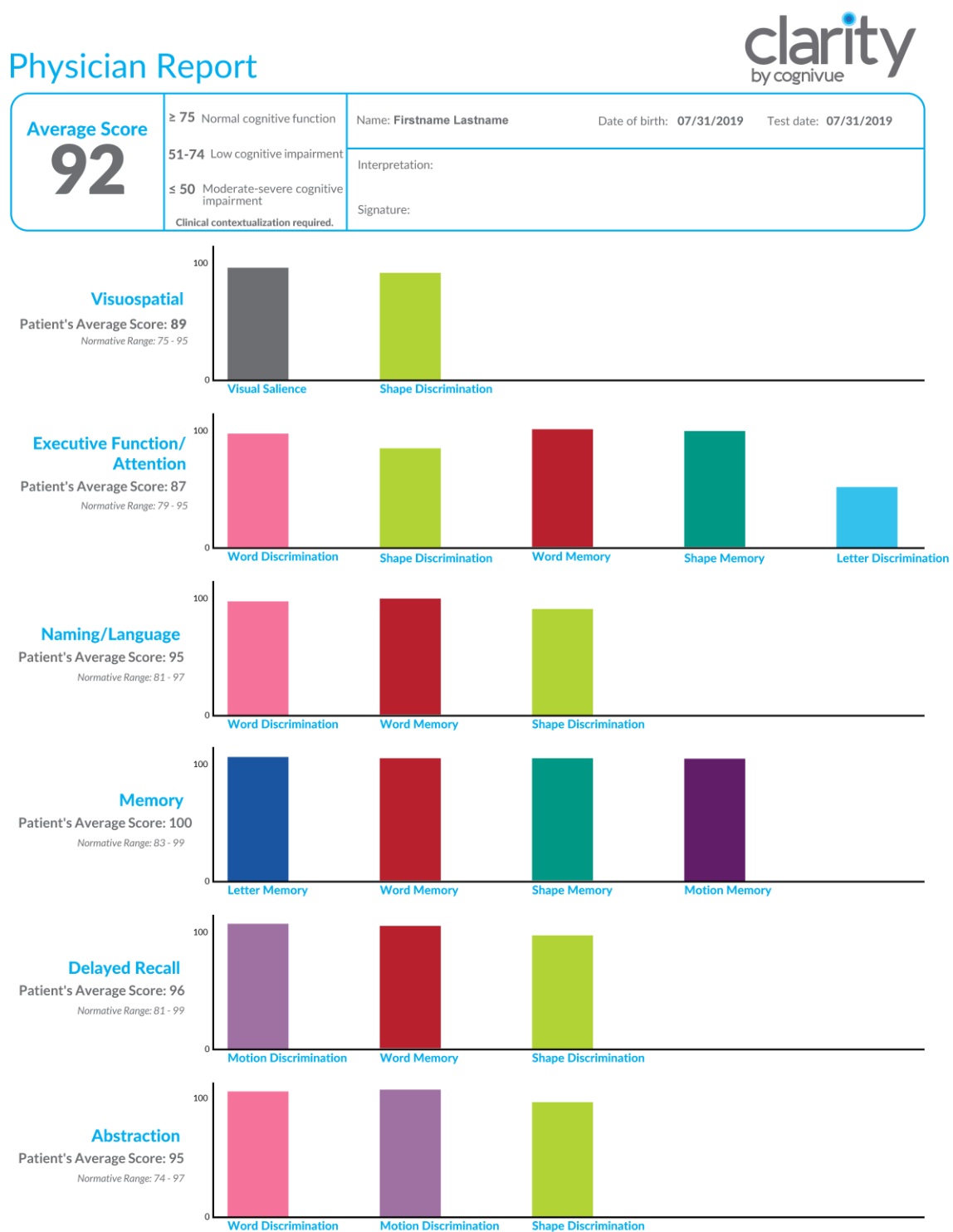

Supplement: Supplementary file 2 [file medi-101-e31125-s002.pdf]

Supplemental digital content 3: Image that illustrates the Clarity Report page 2.

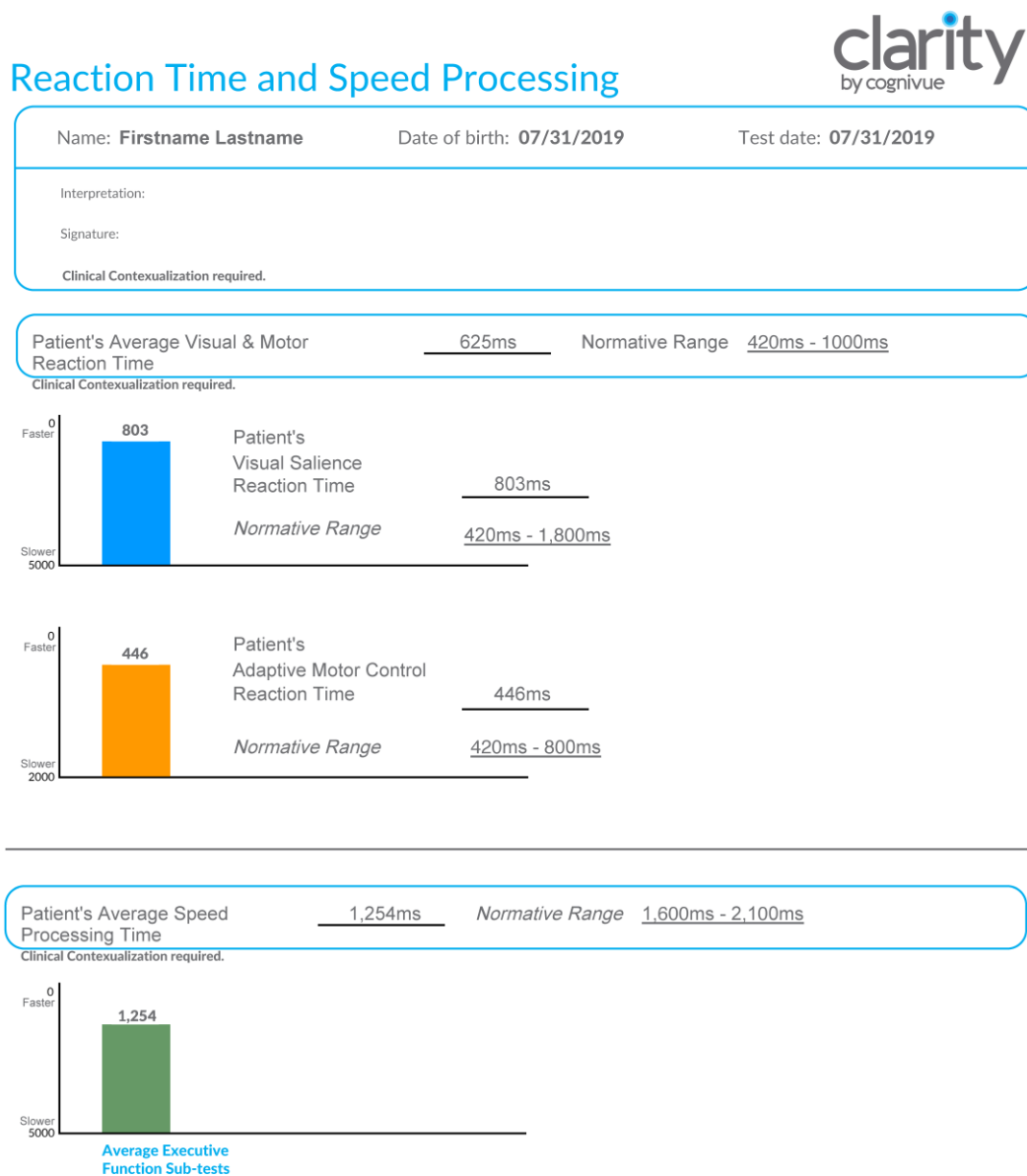

Supplement: Supplementary file 3 [file medi-101-e31125-s003.pdf]
